# Experimental models of epilepsy: A comprehensive review of mechanisms, translational relevance, and future directions

**DOI:** 10.14202/vetworld.2025.3041-3050

**Published:** 2025-10-14

**Authors:** P. J. Jiji, Rajalakshmi Rai, Nayanatara Arun Kumar, Vandana Blossom, Mangala M Pai, Ashwin R. Rai, Rajanigandha Vadgaonkar, S. Dhanya Nayak

**Affiliations:** 1 Department of Anatomy, Kasturba Medical College Mangalore, Manipal Academy of Higher Education, Manipal, India; 2 Department of Physiology, Kasturba Medical College Mangalore, Manipal Academy of Higher Education, Manipal, India

**Keywords:** animal models, antiepileptic drugs, electroencephalography, epilepsy, kainic acid, kindling, pentylenetetrazol, pilocarpine, translational research

## Abstract

Epilepsy is a chronic neurological disorder characterized by recurrent seizures, affecting millions worldwide. Experimental models play a crucial role in understanding the pathophysiology of seizures and in developing novel antiepileptic therapies. This review summarizes the major experimental models of epilepsy, including chemically induced, electrically induced, and genetic approaches. The strengths, limitations, and translational relevance of each model are discussed with particular emphasis on their applicability to human epilepsy subtypes, such as generalized tonic–clonic and temporal lobe epilepsy. Advances in neuroimaging, omics technologies, and artificial intelligence-based analytics are highlighted for their potential to enhance model accuracy and predictive validity. Ethical considerations, including the principles of replacement, reduction, and refinement, are also emphasized. By integrating classical models with emerging technologies, this review provides a comprehensive framework to guide future research aimed at improving therapeutic strategies and bridging the gap between pre-clinical and clinical epilepsy research.

## INTRODUCTION

Epilepsy is a chronic, non-communicable neurological disorder that disrupts normal brain function, affecting nearly 65 million individuals worldwide [1]. It is characterized by sudden, recurrent episodes of involuntary movements involving parts of the body or the entire body, often accompanied by transient loss of consciousness and disturbances in bladder or gastrointestinal control. Seizures can range from brief lapses in responsiveness or minor muscle twitches to severe and prolonged convulsions [1]. These events result from abnormal, excessive electrical discharges in specific brain regions, with frequency varying from less than once per year to multiple episodes within a single day [1]. Electroencephalography (EEG) abnormalities, reflecting both structural and functional brain alterations, remain a primary diagnostic hallmark of epilepsy [2]. Pre-clinical animal studies are indispensable for evaluating the safety and efficacy of antiepileptic drugs (AEDs) before clinical trials [3, 4]. Among these, the choice of an appropriate animal model is a pivotal step in early *in vivo* drug testing and development [5], with rodents being the most extensively employed species in experimental epilepsy research [3, 4].

Although a wide range of experimental epilepsy models have been developed, none fully replicate the complexity and heterogeneity of human epilepsies. Classical chemically induced models, such as pentylenetetrazol (PTZ), kainic acid (KA), and pilocarpine, have been instrumental in elucidating seizure mechanisms and screening antiseizure drugs, yet they often exhibit high mortality, inconsistent pathology, or limited chronicity. Electrical models, including maximal electroshock and kindling, allow precise control of seizure induction but fail to reproduce spontaneous seizures and the broader neuropsychiatric comorbidities commonly seen in patients. Genetic models, while offering insights into inherited epilepsies such as Dravet syndrome, remain resource-intensive, strain-specific, and less representative of acquired epilepsies. Furthermore, most existing models inadequately capture pharmacoresistance, neuroinflammation, and cognitive or behavioral deficits that profoundly impact patients’ quality of life. Recent advances in imaging, omics technologies, and artificial intelligence (AI) promise to refine these models, yet systematic integration of such innovations into pre-clinical epilepsy research remains limited. This gap underscores the need for a critical evaluation of existing models and identification of strategies to enhance their translational relevance.

The aim of this review is to provide a comprehensive evaluation of experimental epilepsy models, with emphasis on their underlying mechanisms, clinical relevance, and limitations. Specifically, this study seeks to (i) compare chemical, electrical, and genetic models in terms of their ability to replicate human seizure phenotypes; (ii) highlight the strengths and drawbacks of each model for pre-clinical AED development; and (iii) explore how emerging approaches, including neuroimaging, metabolomics, proteomics, and AI-driven analytics, can improve model accuracy, predictive validity, and reproducibility. By synthesizing classical methodologies with modern technological innovations, this review aims to outline future directions for developing ethically sound, multimodal, and clinically relevant models that can bridge the gap between bench research and clinical application in epilepsy management.

## ETHICAL CONSIDERATIONS AND WELFARE OF ANIMALS

### Institutional approval and compliance

All animal experiments must be conducted in strict accordance with institutional and international ethical guidelines. Prior approval from the Institutional Ethics Committee is mandatory before initiating any experimental protocol.

### Animal housing and care

Animals should be maintained under appropriate housing conditions with environmental enrichment and continuous care to minimize stress. Welfare considerations extend throughout the study duration to ensure scientific reliability and humane treatment.

### Minimizing pain and distress

Epilepsy induction protocols must be designed to minimize animal suffering. The use of appropriate anesthetics and analgesics is essential to reduce procedural pain and distress.

### Application of the replacement, reduction, and refinement (3Rs)

The principles of 3Rs should be central to experimental design to balance scientific validity with ethical responsibility [5].

### Training and monitoring

Personnel involved in animal handling must be adequately trained to recognize seizure severity, behavioral distress, and early signs of complications. This ensures timely interventions and adherence to welfare standards [5].

## CHEMICALLY INDUCED EPILEPSY MODELS

Chemically induced models provide critical insights into seizure pathophysiology by manipulating seizure thresholds and mimicking various seizure phenotypes. Common agents such as KA and pilocarpine have been widely employed to replicate both acute seizures and chronic epileptic states, often accompanied by neurodegeneration [6]. These models are especially valuable for testing AED efficacy, exploring novel therapeutics, and studying temporal lobe epilepsy (TLE), thereby improving translational relevance [6–8].

### PTZ model

The PTZ model, based on antagonism of Gamma-Aminobutyric acid sub-type A receptors (GABA_A_R)receptors, is among the most widely used chemically induced epilepsy models [7–9]. By blocking inhibitory GABAergic transmission, PTZ induces neuronal hyperexcitability and seizure activity through enhanced glutamatergic signaling.

#### Types of PTZ models

- Acute model: Seizures are induced by single PTZ doses through various injection routes, with severity assessed using scales such as Racine’s [10].
- Chronic kindling model: Repeated subconvulsive doses gradually induce long-term seizure susceptibility, closely mimicking generalized tonic–clonic seizures in humans [11].

#### Protocols

- Daily dosing: Subconvulsive PTZ injections over 6–8 weeks (29–36 injections) until kindling is achieved [12].
- Alternate-day dosing: Subconvulsive doses every 48 h, requiring 13–19 injections over 4–6 weeks, sometimes followed by a challenge dose [10].
- Hybrid protocol: Initial four subconvulsive doses, followed by a rest period, and then daily injections for 3 days (25 days total) [10].

#### Pharmacological control

Sodium valproate, a standard AED, is commonly used as a positive control in PTZ models, with effective doses around 300 mg/kg [13, 14].

### KA model

The KA model is a well-established paradigm for TLE [15]. KA, a glutamate analog derived from red algae, acts as an excitotoxic agent, causing hippocampal neurodegeneration and chronic epileptic states [16, 17].

#### Mechanism and pathology

KA induces hyperexcitability and cell death, particularly in hippocampal pyramidal neurons. Classic studies demonstrated that both intraventricular and intra-amygdaloid injections lead to hippocampal neurodegeneration and seizure activity [18, 19]. Similar pathology has been replicated in non-human primates [20].

#### Routes of administration

- Direct intracerebral injections: Targeted induction but invasive, requiring surgery.
- Systemic administration: Non-invasive, allows simultaneous treatment of multiple animals, but introduces variability in bioavailability and seizure outcomes [21].

#### Limitations

Mortality rates vary widely (5%–30%) depending on dose and route. Typically, a single systemic dose of 6–15 mg/kg is sufficient to induce status epilepticus (SE) in rodents [22, 23].

### Pilocarpine model

The pilocarpine-induced epilepsy model shares several electroencephalographic and neuropathological features with the KA model [24]. Although the extensive neuronal damage caused by pilocarpine is sometimes regarded as a limitation, it closely mirrors the extrahippocampal pathology observed in patients with TLE. A distinguishing feature of pilocarpine is its ability to increase blood–brain barrier permeability, a process thought to facilitate seizure initiation, similar to KA-induced effects [25, 26].

The pilocarpine model is considered highly reliable and efficient, as most animals develop spontaneous recurrent seizures following a single administration or after one to two supplemental doses [27]. Compared to KA, pilocarpine generally establishes chronic epilepsy more rapidly and consistently, reducing the need for multiple injections and minimizing latency to seizure onset.

In contrast, the electrical kindling model requires repeated subconvulsive electrical stimulations of targeted brain regions to gradually induce chronic hyperexcitability and spontaneous seizure activity. The primary advantage of kindling lies in its precise stimulation control, allowing researchers to localize seizure activity and adjust induction parameters [26, 27]. However, unlike KA and pilocarpine models, kindling does not inherently produce overt brain lesions. Structural alterations, such as focal neuronal loss, typically occur only after multiple stage 5 seizures, and hippocampal sclerosis characteristic of human TLE is not replicated. Moreover, kindling is time-intensive and may fail to consistently generate spontaneous seizures or chronic pathology, limiting its translational applicability for TLE research.

### Flurothyl model

Flurothyl, an inhaled convulsant agent, induces seizures by disrupting neuronal excitability, likely through modulation of GABA receptor activity. It primarily elicits generalized tonic–clonic seizures, making it a valuable tool for screening the efficacy of anticonvulsant drugs [28]. Because of its rapid onset and reproducibility, the flurothyl model is especially useful in evaluating short-term pharmacological responses in acute seizure studies, particularly for generalized seizure activity [28].

### Organophosphate model

The organophosphate model, most commonly represented by soman, induces seizures through acetylcholinesterase inhibition, which elevates acetylcholine levels in the brain and triggers excessive neuronal excitation [29]. Seizures are typically generalized and can progress to SE.

This model is widely employed to investigate the neurotoxic effects of organophosphates and to test potential countermeasures against chemical warfare agents. Beyond toxicological applications, it also serves as a robust paradigm for studying SE and its long-term neurological consequences, including cognitive decline and neurodegeneration [30].

## MODELS OF ELECTRICALLY INDUCED EPILEPSY

### Maximal electroshock seizure (MES) model

The MES test is one of the most widely used pre-clinical models for assessing the efficacy of AEDs, particularly against generalized tonic–clonic seizures [31]. In this model, seizures are induced by applying an electrical stimulus to rodents.

The MES model is valued for its simplicity, reproducibility, and minimal technical requirements, making it a reliable tool in anticonvulsant drug screening [31]. It is particularly useful for identifying compounds that block voltage-gated sodium channels (e.g., carbamazepine, phenytoin) or influence neurotransmission through GABAergic enhancement or glutamatergic inhibition [32].

In conventional protocols, the electrical stimulus is set at 5–10 times above the seizure threshold to ensure consistency and reduce variability [31, 32]. Stimulation is typically delivered via corneal or ear-clip electrodes. Standard parameters include currents of 50–150 mA in mice and 150 mA in rats, with a pulse frequency of 50–60 Hz, pulse width of 0.6 ms, and a duration of 0.2 s. Corneal electrodes are often preferred due to improved electrode–skin contact and ease of use.

Seizures induced by MES progress through distinct phases: An immediate tonic phase characterized by hind limb extension, followed by clonic movements such as limb paddling and tremors. Recovery generally occurs within 20–30 s. A seizure is classified as “maximal” if tonic hind limb extension persists for more than 3 s with limbs extended at least 90° from the body. A positive MES response is confirmed when untreated animals consistently exhibit tonic hind limb extension in three independent trials on separate days [33].

### MES threshold (MEST) model

One limitation of the conventional MES test is its reliance on supramaximal stimulation, which can overlook drugs that modestly increase seizure thresholds without blocking high-intensity seizures. For example, ethosuximide, which effectively elevates seizure thresholds, may appear inactive in the MES test despite proven clinical relevance [34].

The MEST model addresses this limitation by using variable current intensities while keeping the drug dose constant. This approach allows precise determination of each animal’s seizure threshold and provides a more sensitive assessment of anticonvulsant efficacy [3].

The MEST model is particularly valuable for evaluating GABAergic agents and compounds that would otherwise be missed in conventional MES assays. However, because seizure thresholds can fluctuate with age, circadian rhythms, and hormonal variations, daily vehicle-treated control groups are essential for accurate comparisons. This requirement adds complexity compared to the standard MES method but significantly enhances sensitivity and translational relevance [3].

### Electrical kindling model

The electrical kindling model involves the repeated administration of subthreshold electrical stimuli to specific brain regions, leading to a progressive and permanent increase in seizure susceptibility [35]. This model effectively simulates the gradual development of epilepsy, particularly TLE.

Key advantages of kindling include its reproducibility, ability to mimic epileptogenesis, and suitability for studying long-term AED efficacy. Furthermore, it allows precise experimental control over stimulation sites and parameters, with both slow and rapid kindling protocols available to modulate the induction timeline.

Despite its strengths, kindling has limitations. The model is surgically invasive, requiring electrode implantation, and time-consuming, especially under traditional protocols. Moreover, it is less suitable for modeling generalized or acute-onset seizures, and in many cases, it does not consistently generate spontaneous recurrent seizures. Nonetheless, the electrical kindling model remains a cornerstone of chronic epilepsy research, particularly for elucidating the mechanisms of epileptogenesis and seizure progression [36].

## GENETIC EPILEPSY MODELS

Genetic models of epilepsy, such as sodium channel protein type 1 subunit alpha (SCN1A) knockout mice, provide critical insights into the mechanisms underlying inherited epileptic syndromes, including Dravet syndrome [37]. These models are invaluable for exploring the molecular and cellular basis of epileptogenesis, identifying genetic contributors, and evaluating targeted therapeutic interventions. While their primary utility lies in mechanistic and molecular studies, they also serve as important tools for pre-clinical screening of precision-based therapies.

Genetic approaches complement traditional chemical and electrical seizure models, which remain fundamental in the development of broad-spectrum AEDs [38]. For example, the maximal electroshock (MES) model continues to be highly effective in evaluating sodium channel blockers such as phenytoin and carbamazepine, underscoring its role in the study of generalized tonic–clonic seizures [39]. Similarly, the PTZ model remains widely employed to assess the efficacy of GABAergic drugs, including benzodiazepines and valproate [40, 41].

Furthermore, models such as pilocarpine and KA, which closely mimic the neuropathological features of TLE, are particularly suited for testing antiglutamatergic compounds, neuroprotective agents, and potential disease-modifying therapies. Thus, while genetic models advance understanding of inherited epilepsies, the integration of genetic, chemical, and electrical models provides a comprehensive pre-clinical framework for evaluating novel therapeutic strategies across the spectrum of epileptic disorders. Comparison of various experimental epilepsy models in animals has been summarized in Table 1 [12, 15, 25, 28, 29, 31, 35, 37].

**Table 1:** Comparison of experimental epilepsy models in animals.

Model and reference	Induction method	Modeled seizure type	Translational relevance	Advantages	Limitations
Pentylenetetrazol [12]	Chemical (GABA_A_ receptor antagonist)	Generalized tonic–clonic and myoclonic	Moderate	Simple, reproducible, and suitable for screening GABAergic drugs	Limited in modeling chronic epilepsy and its complex features
Kainic acid [15]	Chemical (glutamate analog)	TLE	High	Mimics hippocampal damage, useful for chronic studies and neurodegeneration	High variability in mortality outcomes
Pilocarpine [25]	Chemical (muscarinic receptor agonist)	TLE, status epilepticus	High	Reproducible TLE model with comorbidities	Widespread brain damage and systemic toxicity
Flurothyl [28]	Chemical (inhaled convulsant)	Generalized tonic–clonic	Moderate	Non-invasive, rapid induction, suitable for acute screening	Requires a specialized setup
Organophosphate [29]	Chemical (AchE inhibitor)	Generalized seizures (SE)	Moderate	Relevant to toxicology and SE research	High systemic toxicity and ethical and safety concerns
MES [31]	Electrical stimulation (supramaximal stimulation)	Generalized tonic–clonic	High	Standard model for screening sodium channel blockers	Not suitable for drugs acting on absence or partial seizures
MES [31]	Electrical (threshold detection)	Generalized seizures	Moderate	Detects subtle anticonvulsant effects and is sensitive to GABAergic agents	More complex setup and day-to-day variability
Electrical kindling [35]	Repeated focal subthreshold electrical stimulus	Focal and secondary generalized	High	Mimics progressive epileptogenesis and comorbidities	Time-consuming and may not always produce spontaneous seizures
Sodium channel protein type 1 subunit alpha knockout [37]	Genetic mutation or knockout	Generalized, febrile, and spontaneous	Very high	Models of pharmacoresistant epilepsy and genetic mechanisms	Expensive, strain-specific, limited to inherited epilepsy

TLE = Temporal lobe epilepsy, SE = Status epilepticus, MES = Maximal electroshock seizure

## INTEGRATION OF BEHAVIORAL AND EEG ASSESSMENTS

### EEG and behavioral correlation

The integration of EEG with behavioral scoring systems, such as the Racine scale, provides a robust framework for classifying and quantifying seizure severity. This combined approach enables researchers to correlate electrophysiological abnormalities with observable seizure behaviors, ensuring accurate identification of seizure type, frequency, and progression across both acute and chronic stages.

### Applications in chronic epilepsy models

Chronic epilepsy models, such as kindling and pilocarpine-induced epilepsy, extend beyond seizure generation to encompass clinically relevant comorbidities. These models allow researchers to study epilepsy-associated depression, cognitive impairment, and neuroinflammation, thereby offering a translational platform for evaluating therapeutic strategies that target both seizures and their neuropsychiatric consequences [42].

## COMORBIDITIES AND EXTENDED OUTCOMES

### Neuropsychiatric dimensions of epilepsy

Epilepsy is frequently associated with comorbid neuropsychiatric conditions, including depression, anxiety, cognitive impairment, and neuroinflammation. These comorbidities substantially affect patient quality of life and treatment outcomes.

### Limitations of acute models

Traditional acute seizure models often fail to capture these extended pathologies, limiting their clinical relevance.

### Value of chronic models

Chronic models, such as pilocarpine, KA, and electrical kindling, are increasingly used to simulate long-term disease progression and associated comorbidities. These models enable the simultaneous evaluation of behavioral, cognitive, and emotional impairments alongside seizure activity, thus providing a more comprehensive and clinically relevant framework for testing novel therapeutics. Incorporating such dimensions into experimental design enhances the translational value of pre-clinical epilepsy research.

## ADVANCED TECHNOLOGIES ENHANCING THE UTILITY OF MODELS

### Neuroimaging and AI integration

Recent advances in technology are transforming pre-clinical epilepsy research by offering more accurate tools to study seizure mechanisms and therapeutic responses. Modern imaging modalities such as magnetic resonance imaging (MRI), positron emission tomography, and *in vivo* calcium imaging allow detailed visualization of structural and functional brain alterations associated with epilepsy [43].

Parallel advances in AI are enabling seizure forecasting, automated behavioral monitoring, and integration of large, complex datasets. These innovations improve the accuracy, reproducibility, and translational relevance of pre-clinical models.

### Omics approaches

Metabolomics is emerging as a powerful strategy for identifying biomarkers of drug response. Analysis of blood, cerebrospinal fluid, or brain tissue can reveal biochemical differences between responders and non-responders to antiepileptic therapy [44]. When integrated with genomics and proteomics, metabolomics enhances the ability to unravel the multilayered biological pathways underlying drug response, paving the way for personalized epilepsy treatment strategies.

### EEG with real-time imaging and AI

The combination of EEG with functional MRI, calcium imaging, or optogenetics provides a multidimensional view of seizure dynamics. When augmented with AI algorithms, these approaches enable precise spatiotemporal mapping of seizure onset and propagation, aiding in the identification of epileptogenic zones [45].

Moreover, AI-driven behavioral monitoring systems, powered by deep learning and computer vision, allow automated, real-time seizure detection and classification. These systems can track subtle motor and non-motor seizures with high temporal resolution, reducing observer bias, improving reproducibility, and enabling continuous long-term monitoring in naturalistic environments [46].

## COMPARATIVE LIMITATIONS OF EPILEPSY MODELS AND FUTURE REFINEMENTS

### Inherent limitations of existing models

Despite the diversity of experimental epilepsy models, none fully replicates the complexity and heterogeneity of human epilepsies. Each model carries inherent limitations that must be acknowledged when translating pre-clinical findings to clinical practice [47, 48].

- Chemical models (PTZ, KA, pilocarpine): Although cost-effective and reproducible, they often induce widespread neuronal damage and fail to mimic the focal pathology observed in many human epilepsies. KA and pilocarpine, while closer to TLE, exhibit variability in seizure onset, mortality rates, and extent of neurodegeneration.
- Electrical models (MES, kindling): The MES model is widely used for screening sodium channel blockers but does not simulate spontaneous seizures or underlying structural changes. Electrical kindling offers excellent control and reproducibility but requires prolonged stimulation and may not always result in spontaneous seizures. Both models lack representation of inflammatory and neurodegenerative processes.
- Genetic models: Mouse models such as SCN1A knockouts provide critical insights into inherited epilepsies but often fail to capture the acquired nature of most human cases. They require complex breeding and may not consistently exhibit spontaneous seizures.
- Other toxin-induced models (soman, flurothyl): These are highly specific but raise safety and ethical concerns. Flurothyl is limited to modeling acute seizures, while organophosphate models often result in severe systemic toxicity that can overshadow seizure-related outcomes.

### Directions for refinement

**Figure 1:**
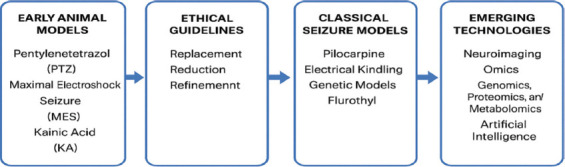
Pre-clinical seizure models and integration of emerging epilepsy research technologies.

Future refinements should focus on:

- Developing models with greater subtype specificity, such as those targeting drug-resistant focal epilepsy and epileptic encephalopathies.
- Incorporating common comorbidities (e.g., cognitive impairment, mood disorders) to better reflect clinical reality.
- Employing humanized systems and patient-derived induced pluripotent stem cells (iPSCs) for enhanced genetic and molecular relevance.
- Integrating multimodal approaches that combine behavioral assessments, EEG, neuroimaging, and omics technologies.
- Adopting AI-driven monitoring and 3Rs-based ethical practices to improve reproducibility and humane standards [49; Figure 1].

## FUTURE PERSPECTIVES AND MODEL REFINEMENTS

### Personalization and subtype-specific models

The future of epilepsy modeling lies in creating personalized and subtype-specific models. These should account for disease heterogeneity, with a particular focus on drug-resistant and genetic epilepsies. Incorporating neuropsychiatric comorbidities, such as depression and cognitive decline, into chronic epilepsy models will enhance their clinical relevance.

### Emerging pre-clinical tools

Cutting-edge platforms are advancing pre-clinical epilepsy research:

- Humanized mouse models and patient-derived iPSCs provide genetically faithful systems.
- Non-mammalian models such as zebrafish enable high-throughput screening while offering transparency for imaging studies.
- Integrative omics (genomics, proteomics, metabolomics) combined with neurotechnology (EEG, imaging, AI-based analysis) can deliver comprehensive insights into seizure mechanisms and therapeutic responses.

### Bridging the translational gap

The clinical failure of promising drugs (e.g., retigabine) highlights the limitations of current pre-clinical approaches and underscores the need for models that replicate pharmacoresistance, chronicity, and comorbidities. Future research should emphasize precision-matched therapies, individualized seizure prediction, and multimodal data integration.

Collectively, these advancements are expected to strengthen the translational bridge between bench and bedside, ultimately paving the way for personalized epilepsy treatments and improved outcomes for patients with refractory epilepsy.

## CONCLUSION

This review highlights the diverse range of experimental epilepsy models, including chemical, electrical, and genetic approaches, each offering unique contributions to understanding epileptogenesis and evaluating therapeutic strategies. Chemical models such as PTZ, KA, and pilocarpine have been instrumental in replicating seizure phenotypes and testing AEDs, while electrical models such as MES and kindling remain indispensable for mechanistic studies and drug screening. Genetic models, such as SCN1A knockouts, have advanced knowledge of inherited epilepsies. Integration of EEG with behavioral scoring systems has improved seizure classification, and chronic models have enabled investigations into comorbidities including depression and cognitive decline. Emerging technologies such as omics-based profiling, advanced imaging, and AI-driven monitoring are transforming pre-clinical epilepsy research by enhancing accuracy, reproducibility, and translational value.

These models collectively underpin the discovery, refinement, and validation of antiepileptic therapies, guiding drug safety and efficacy evaluations and offering translational frameworks for bridging pre-clinical findings to clinical application. Their strength lies in their reproducibility, standardization, and ability to test targeted mechanisms as well as in their increasing integration with multimodal tools. However, no single model fully captures the heterogeneity, chronicity, and comorbidities of human epilepsy. Chemical models often induce widespread neuronal damage, electrical models lack spontaneous seizures and inflammatory pathology, genetic models are resource intensive, and toxin-based models such as flurothyl and organophosphates pose safety and ethical concerns. These limitations highlight the need for cautious interpretation when extrapolating findings to human disease.

Future refinements should focus on the development of subtype-specific and humanized models for drug-resistant epilepsies, epileptic encephalopathies, and genetic syndromes. Patient-derived iPSCs, humanized mouse systems, and zebrafish platforms offer promise for precision research and high-throughput testing. The integration of genomics, proteomics, metabolomics, and AI-based analytics will enable individualized seizure prediction and tailored therapeutic strategies. Ethical practices guided by the principles of 3Rs will further strengthen the reproducibility and humane standards of pre-clinical research.

In conclusion, experimental epilepsy models remain essential for unraveling disease mechanisms and developing therapies, but refinement is crucial to enhance clinical translation. A multimodal, ethically grounded, and clinically aligned approach that merges traditional models with emerging technologies holds the potential to accelerate next-generation therapies and advance personalized medicine, ultimately improving outcomes for patients with epilepsy, particularly those with drug-resistant forms.

## AUTHORS’ CONTRIBUTIONS

PJJ and NAK: Designed the study and drafted the manuscript. RR, VB, ARR, MMP, RV, and SDN: Reviewed the manuscript. All authors have read and approved the final manuscript.
